# Prevalence of Epstein-Barr virus DNA and *Porphyromonas gingivalis* in Japanese peri-implantitis patients

**DOI:** 10.1186/s12903-017-0438-6

**Published:** 2017-12-12

**Authors:** Ayako Kato, Kenichi Imai, Hiroki Sato, Yorimasa Ogata

**Affiliations:** 1 Yorimasa Ogata, Department of Periodontology and Research Institute of Oral Science, Nihon University School of Dentistry at Matsudo, 2-870-1 Skakaecho-nishi, Matsudo, Chiba, 271-8587 Japan; 2 Department of Microbiology, Division of Immunology and Pathobiology, Dental Research Center, Nihon University School of Dentistry, Tokyo, 102-8310 Japan

**Keywords:** Epstein-Barr virus, *Porphyromonas gingivalis*, Peri-implantitis, Prevalence

## Abstract

**Background:**

Peri-implantitis (PI) is an inflammatory reaction associated with functional deterioration of supporting bones around the dental implant. Recent studies suggested Epstein–Barr virus (EBV) is involved in the pathogenesis of periodontitis. We investigated the association between EBV and *Porphyromonas gingivalis* in Japanese PI patients.

**Methods:**

Fifteen periodontally healthy individuals, 15 healthy implant patients and 15 PI patients were recruited. Forty five subgingival plaque samples were collected from the deepest probing pocket depth (PPD) site from each patient. Real-time PCR was used to detect EBV DNA and *P. gingivalis*.

**Results:**

EBV and *P. gingivalis* were detected in 7 and 3 PPD sites of the healthy controls, in 9 and 4 PPD sites of the healthy implants, and in 13 and 14 PPD sites of the PI patients. *P. gingivalis* and coexistence of EBV and *P. gingivalis* were detected significantly higher in the PI patients than healthy controls and healthy implant patients. EBV was detected significantly higher in the PI patients than healthy controls.

**Conclusions:**

Higher levels of EBV and *P. gingivalis* were detected in PPD sites of PI patients. These results suggest that coexistence of EBV and *P. gingivalis* may serve pathogenic factors cause for PI in Japanese dental patients.

## Background

The Epstein-Barr virus (EBV) is an enveloped herpes virus with double-stranded DNA [1]. EBV is one of the most common viruses, infecting more than 90% of the adult population worldwide [2, 3]. EBV infection is usually asymptomatic during childhood, but the infection leads to infectious mononucleosis when it is delayed until adolescence. It is transmitted from host to host by salivary contact, and the virus passes through the oropharyngeal epithelium to B lymphocytes, where it establishes a lifelong latent infection [4–6]. EBV is associated with Burkitt’s lymphoma, Hodgkin’s lymphoma, natural killer/T-cell lymphoma, post transplantation lymphoproliferative disorder and nasopharyngeal cancer [7–9]. EBV is adsorbed to CD21 receptors which are expressed on B lymphocyte [10–12]. The reactivation of EBV from latent infection occurs frequently and multiplies with the epithelium cells of the pharyngeal and is exhausted in saliva [13].

Association between EBV infection and periodontitis has been reported [14, 15]. Therefore, we have studied the relationship between EBV and *Porphyromonas gingivalis* which is representative periodontal disease pathogen in chronic periodontitis (CP). EBV DNA and *P. gingivalis* were detected in deeper periodontal pockets of Japanese CP patients [16, 17]. Our findings suggest that EBV DNA may serve as a pathogenic factor leading to CP. We also provide evidence for potential interactions between EBV and *P. gingivalis* in the etiopathogenesis of CP [17]. A systematic review indicated that herpesviruses including EBV are significantly associated with CP [18]. Lu et al. [19] reported an EBV and *P. gingivalis* coinfection may promote the development of CP among pregnant women.

Peri-implantitis (PI) is an inflammatory reaction associated with functional deterioration of supporting bones around dental implants [20]. PI is the most frequent long-term complications of dental implants [20–24]. It provokes bone destruction with suppuration (SUP), and encountered swelling and bleeding on probing (BOP) around implants. Clinical manifestations characterized by the destruction of bone are similar to periodontitis. Several studies showed that the periodontopathic bacteria are detected in PI sites and that these bacteria are also detected in periodontitis [25–28]. On the other hand, several studies suggest that the bacteria flora from PI sites were different from periodontitis sites, or tend to create ecologically different bacterial communities between PI and periodontitis [29–32].

Therefore, the purpose of this study was to examine whether EBV DNA and *P. gingivalis* are associated with deep pockets in Japanese PI patients.

## Methods

### Subjects and subject distribution

Fifteen periodontally healthy individuals (healthy controls: mean age, 55.5 ± 14.8 years), 15 healthy implant patients (mean age, 60.7 ± 10.0 years) and 15 PI patients (mean age, 63.9 ± 12.7 years) were included in this study. They received periodontal initial treatment at Nihon University Hospital School of Dentistry at Matsudo, Japan. The Institutional Review Board at the Nihon University School of Dentistry at Matsudo approved the study (EC14–11–027-1, EC15–005). Written informed consent was obtained from each study subject after all experiments were fully explained. Diagnosis of PI was defined as changes in the level of the crestal bone on radiographic examination in conjunction with BOP, with or without concomitant deepening of peri-implant pockets [33]. SUP is a common finding in PI sites. Healthy implant sites showed no clinical signs of peri-implant mucositis or PI. A group of 15 individuals without periodontitis were included as the healthy control group. The healthy controls showed no clinical signs of gingivitis or attachment loss, no detectable bone loss on radiographic examination, and a probing pocket depth (PPD) of ≤3 mm. All samples were taken from the deepest PPD site per patient (15 healthy controls, 15 healthy implant patients, and 15 PI patients). All patients were systemically healthy and had no history of periodontal treatment or any type of antibiotic therapy for at least three months prior to the present study.

### Sampling

Fifteen subgingival plaque samples were collected from one periodontally healthy site of PPD (≤3 mm) among 15 healthy controls, and 15 subgingival plaque samples were collected from one healthy implant site of PPD (≤4 mm) among 15 healthy implant patients, and 15 subgingival plaque samples were collected from one PI site of PPD (≥5 mm) among 15 PI patients. Before sampling, supragingival plaque was removed with Gracey curette. Sterile paper points were inserted to the sample site (three times), retained for 30 s, pooled in Eppendorf tubes, and then stored at −80 °C [17].

### DNA extraction and real-time PCR

DNA samples from the subgingival plaque were prepared using High Pure Viral Nucleic Acid Kit (Roche Applied Science, Mannheim, Germany). Quantitative real-time polymerase chain reaction (PCR) was used to measure the copy numbers of EBV DNA and *P. gingivalis* in the samples, using the following primer sets: EBV forward, 5′-CCTGGTCATCCTTTGCCA-3′; EBV reverse, 5′-TGCTTCGTTATAGCCGTAGT-3′; *P. gingivalis* forward, 5′-AGGCAGCTTGCCATACTGCG-3′; *P. gingivalis* reverse, 5′-ACTGTTAGCAACTACCGATGT-3′; glyceraldehyde-3-phosphate dehydrogenase (GAPDH) forward, 5′-GCACCGTCAAGGCTGAGAAC-3′; and GAPDH reverse, 5′-ATGGTGGTGAAGACGCCAGT-3′ [17]. SYBR Premix Ex Taq in a TP800 thermal cycler dice real-time system (Takara-bio, Tokyo, Japan) was used for real-time PCR reaction in a total volume of 25 μl (12.5 μl 2× SYBR Premix Ex Taq, 0.2 μl forward and reverse primers, and 12.1 μl as the DNA sample (300 ng per 1 well) and the PCR products comprised of 95 bp for EBV, 404 bp for *P. gingivalis*, and 142 bp for GAPDH. The thermal cycling conditions were 10 s at 95 °C, 45 cycles of 5 s at 95 °C and 30 s at 60 °C. The dynamic ranges of the real-time PCR assays were determined through serial dilution of DNA extracts either as AKATA cells or *P. gingivalis* TDC60 of the standards in the range of 10^9^ ∼ 10^1^copies/ml [34, 35].

### Statistical analysis

The chi squared test for independence, confirmed by Fisher’s exact probability test, was used to determine whether individual pathogens were associated with PI, and to calculate the odds ratio. *P values* of <0.05 were considered to be statistically significant.

## Results

The age, sex, PPD, BOP, SUP, loading time of implants and patient distribution are summarized in Table 1. The mean PPD of the healthy controls was 2.73 ± 0.44 mm (mean ± SD). The average PPD of the healthy implant sites and the PI sites were 2.87 ± 0.62 mm and 6.20 ± 1.60 mm, respectively. BOP was detected in 1 healthy implant site and 11 PI sites and undetectable in healthy controls. Moreover, SUP was detected in 9 PI sites. Table 2 shows clinical data and counts of EBV DNA and *P. gingivalis* (copies/ml) in the healthy controls. EBV DNA and *P. gingivalis* were detected in 7 of 15 healthy PPD sites (46.7%, range from 9.19 × 10^3^~3.88 × 10^8^ copies/ml) and 3 of 15 healthy PPD sites (20.0%, range from 9.17 × 10^6^~1.31 × 10^9^ copies/ml). Table 3 describes clinical data and counts of EBV DNA and *P. gingivalis* in the healthy implants. EBV DNA and *P. gingivalis* were detected in 9 of 15 healthy implant sites (60.0%, range from 1.71 × 10^4^~2.97 × 10^8^ copies/ml), and 4 of 15 healthy implant sites (26.7%, 2.38 × 10^7^~6.79 × 10^10^ copies/ml). Table 4 shows clinical data and counts of EBV DNA and *P. gingivalis* in the PI sites. EBV DNA and *P. gingivalis* were detected in 13 of 15 PI sites (86.7%, range from 1.06 × 10^4^~4.79 × 10^9^ copies/ml), and 14 of 15 PI sites (93.3%, range from 5.4 × 10^6^ ~ 4.42 × 10^10^ copies/ml). Loading time of healthy implant group and PI group were 4.45 ± 2.53 years (mean ± SD) and 5.63 ± 2.72 years, respectively (Table 1). Mean loading time of PI group was longer than healthy implant group. However, there was no significant difference between two groups.

**Table 1:** Characteristics of subjects and clinical data in this study

	HC	HI	PI
	(15 healthy controls)	(15 healthy implants)	(15 peri-implantitis)
Age	55.5 ± 14.8	60.7 ± 10.0	63.9 ± 12.7
Males	5 (33.3%)	4 (26.7%)	9 (60%)
Females	10 (66.7%)	11 (73.3%)	6 (40%)
PPD (mm)	2.73 ± 0.44	2.87 ± 0.62	6.20 ± 1.60
BOP	0 (0%)	1 (6.7%)	11 (73%)
SUP	0 (0%)	0 (0%)	9 (60%)
Loading time (y)	**−**	4.45 ± 2.53	5.63 ± 2.72

Probing pocket depth (PPD), Bleeding on probing (BOP), Suppuration (SUP)

**Table 2:** Clinical data and counts of EBV and *P. gingivalis* in the healthy controls

Subject	Gender	Age	PPD	BOP	EBV	*P. gingivalis*
no.			(mm)		(copies/ml)	(copies/ml)
1	Female	73	2	–	8.44×10^6^	ND
2	Female	60	3	–	ND	ND
3	Female	72	3	–	ND	ND
4	Male	60	2	–	8.00×10^4^	ND
5	Female	60	2	–	9.19×10³	ND
6	Female	42	3	–	3.64×10^5^	ND
7	Female	45	3	–	ND	ND
8	Female	55	3	–	8.65×10^5^	ND
9	Female	74	3	–	9.38×10^5^	ND
10	Male	73	3	–	3.88×10^8^	2.82×10^8^
11	Female	47	3	–	ND	1.31×10^9^
12	Male	26	3	–	ND	ND
13	Male	28	2	–	ND	ND
14	Female	58	3	–	ND	9.17×10^6^
15	Male	59	3	–	ND	ND

Not detectable (ND)

**Table 3:** Clinical data and counts of EBV and *P. gingivalis* in healthy implants

Subject	Gender	Age	Loading	PPD	BOP	EBV	*P. gingivalis*
no.			time (y)	(mm)		(copies/ml)	(copies/ml)
1	Female	73	8.25	3	–	2.97×10^8^	1.61×10^9^
2	Female	44	1	3	–	ND	ND
3	Female	60	5	3	–	1.71×10^4^	ND
4	Male	60	5	3	–	ND	ND
5	Female	72	4.5	4	–	ND	ND
6	Female	60	0.42	3	+	ND	6.79×10^10^
7	Female	45	0.67	2	–	4.40×10^4^	ND
8	Female	61	2.42	2	–	ND	2.38×10^7^
9	Female	55	4.75	3	–	2.95×10^6^	ND
10	Female	74	4	2	–	1.84×10^7^	ND
11	Male	42	6	3	–	2.73×10^8^	3.51×10^9^
12	Male	63	4	4	–	2.93×10^8^	ND
13	Male	65	8	2	–	8.20×10^6^	ND
14	Female	69	4.75	3	–	7.46×10^5^	ND
15	Female	68	8	3	–	ND	ND

Not detectable (ND), y (years)

**Table 4:** Clinical data and counts of EBV and *P. gingivalis* in peri-implantitis sites

Subject	Gender	Age	Loading	PPD	SUP	BOP	EBV	*P. gingivalis*
no.			Time (y)	(mm)			(copies/ml)	(copies/ml)
1	Male	87	5	8	+	+	3.22×10^5^	5.53×10^8^
2	Female	74	8.67	5	+	–	3.54×10^7^	2.09×10^8^
3	Female	72	6	7	+	+	2.79×10^6^	4.57×10^9^
4	Male	63	5.25	9	+	+	2.34×10^6^	1.39×10^9^
5	Female	60	0.5	5	–	–	ND	1.61×10^9^
6	Female	42	6.25	6	+	–	3.98×10^6^	1.55×10^8^
7	Male	80	6	5	+	+	4.14×10^7^	ND
8	Male	60	2	6	–	+	1.06×10^4^	4.42×10^10^
9	Male	63	10	5	–	–	4.79×10^9^	1.56×10^9^
10	Male	67	2	7	+	+	2.42×10^5^	5.00×10^9^
11	Male	79	10	10	–	+	1.93×10^5^	2.11×10^9^
12	Female	62	5.42	5	+	+	2.33×10^5^	1.42×10^8^
13	Female	47	7	5	+	+	1.39×10^5^	5.40×10^6^
14	Male	59	5.17	5	–	+	ND	8.50×10^7^
15	Male	44	5.17	5	–	+	1.32×10^6^	6.41×10^7^

Not detectable (ND), y (years)

The prevalence of EBV DNA and *P. gingivalis* in the healthy controls, healthy implants and PI sites are listed in Table 5. EBV DNA was detected significantly higher in the PPD sites of PI patients (86.7%) than healthy controls (46.7%), but not statistically significant higher than healthy implant patients (60.0%). *P. gingivalis* occurred at significantly higher frequencies in the PPD sites of PI patients (93.3%) than in the PPD sites of healthy controls (20.0%) and healthy implants (26.7%). Coexistence of EBV DNA and *P. gingivalis* was significantly higher in the PPD sites of PI patients (80.0%) than in the PPD sites of healthy controls (6.7%) and healthy implants (13.3%). To calculate the odds ratio of quantitative risk factors for PI, the findings of healthy implants and PI were compared with that of the healthy controls (Table 6). In the PPD sites of healthy implants, the odds ratios for EBV, *P. gingivalis* and the presence of both EBV and *P. gingivalis* were 1.71, 1.45 and 2.15. In the PPD sites of PI patients, higher odds ratios for EBV, *P. gingivalis* and both EBV and *P. gingivalis* were shown as 7.43, 56 and 56, respectively.

**Table 5:** Occurrence of EBV and *P. gingivalis* in the subgingival samples from HC, HI and PI patients

	Detection frequency			Significance		
Infectious agents	HC	HI	PI	HC vs HI	HC vs PI	HI vs PI
	(n=15)	(n=15)	(n=15)	(*P* value)		
EBV	7 (46.7%)	9 (60.0%)	13 (86.7%)	0.3576246	0.0250875*	0.1073796
*P. gingivalis*	3 (20.0%)	4 (26.7 %)	14 (93.3%)	0.5	0.0000579**	0.0002420**
EBV+*P. gingivalis*	1 (6.7 %)	2 (13.3%)	12 (80.0%)	0.5	0.0000579**	0.0003395**

*p<0.05, **p<0.01 statistically significant, healthy controls (HC), healthy implants (HI), peri-implantitis (PI)

**Table 6:** Association between EBV, *P. gingivalis* and HI or PI

	HI	PI
Microorganisms	odds ratio	odds ratio
EBV	1.71	7.43
*P. gingivalis*	1.45	56
EBV + *P. gingivalis*	2.15	56

## Discussion

In this study, we demonstrated for the first time that higher levels of EBV DNA, *P. gingivalis* and coexistence of EBV DNA and *P. gingivalis* were detected in deep PPD sites of Japanese PI patients compared with healthy controls and healthy implant patients. The results suggest that EBV and *P. gingivalis* may serve exacerbating factors caused by PI.

Although periodontopathic bacteria from pocket a reservoirs around periodontally compromised teeth are considered to be associated with for the subgingival plaque around implants in partially edentulous patients [26, 28, 36], it has become increasingly obvious that EBV is involved in the etiology of PI [37–39]. Bacterial activity alone is not sufficient to explain the following clinical characteristics of periodontitis, such as rapid bone resorption with minimal plaque, site specificity, and presence of active and quiescence phase [40, 41]. In this study, we examined whether higher prevalence of EBV DNA and *P. gingivalis* are associated with deeper PPD sites in Japanese PI patients, because several studies suggest that EBV and *P. gingivalis* act synergistically to potentiate periodontal disease progression and tissue destruction [16, 17, 19, 42, 43]. As expected, we detected higher levels of EBV DNA, *P. gingivalis* and coexistence of EBV DNA and *P. gingivalis* in deeper PPD sites of PI. EBV and human cytomegalovirus (HCMV) trigger a release of inflammatory cytokines that have the potential to activate osteoclasts and matrix metalloproteinases activities, impair antibacterial immune mechanisms, and permit the overgrowth of periodontopathic bacteria [41, 44, 45]. EBV may cooperate with *P. gingivalis* in the destruction of periodontal tissues [42, 45]. A significant correlation was found between presence of subgingival EBV and HCMV and clinical parameters of PI and healthy implant sites, and confirms the high prevalence of EBV and HCMV in subgingival plaque of PI sites [37]. PI lesions were 14.2 times and 3 times more likely to harbor EBV than healthy implant sites and saliva, respectively [39]. Verdugo et al. [46] reported saliva may act as a vehicle to transport EBV and periodontal pathogens into the sinus, and cause bone loss after sinus augmentation. Periodontal pathogen and EBV-associated periapical periodontitis might be the source of retrograde infectious PI [47]. Therefore, EBV could be a potential candidate in PI etiopathogenesis.

Gingival epithelial cells of the periodontium are commonly infected with EBV and may serve as an oral reservoir of latent EBV-infected cells, and the base level of epithelial EBV infection is significantly increased in periodontitis patients [48]. We have previously reported that the results of in situ hybridization of EBV-encoded small RNA (EBER) showed a large number of EBV-infected cells were observed in the inflamed gingival connective tissue subjacent to the gingival epithelium [16]. These results also support the high detection frequency of EBV DNA (46.7% and 60%) even in the gingival sulcus of healthy controls and healthy implant patients in the present study (Table 5).

The latent form of EBV can be induced to enter the lytic replication cycle by treatment with various inducers, such as phorbol 12-myristate 13-acetate, anti-immunoglobulin, calcium ionophore, transforming growth factor-β and butyric acid [5, 6, 49]. The EBV BZLF1 gene product ZEBRA is a master regulator of the transition from latency to the lytic replication cycle. Hypoacetylation of histone in the BZLF1 promoter by histone deacetylase (HDAC) is involved in maintaining EBV latency. Culture supernatant of *P. gingivalis* is containing a high concentration of butyric acid which is an inhibitor of HDAC, increased histone acetylation and transcriptional activity of the BZLF1 gene [5, 6]. These findings suggest that periodontitis and PI are risk factors for EBV reactivation in infected individuals.

Our research provides evidence for potential interactions between EBV and *P. gingivalis* in the etiopathogenesis of peri-implantitis. EBV and periodontal pathogen co-existence apparently leads to synergistic effects and exacerbates the progress of periodontitis and peri-implant diseases [39, 45]. EBV-infected periodontitis and PI lesions tend to harbor elevated levels of periodontopathic bacteria. Viral and bacterial co-existences were reported more frequently in deeper PPD sites of CP patients [42, 50]. We have previously reported that coexistence of EBV DNA and *P. gingivalis* was significantly higher in patients with deeper PPD sites (40%) than in those with shallow PPD sites (14%) or healthy controls (13%) [16]. In addition, coexistence of EBV DNA and *P. gingivalis* was significantly higher in the deeper PPD sites of CP patients (68%) than in the PPD sites of the healthy controls (15%) and shallow PPD sites of CP patients (12%) [17]. These results support the opinion that a combined presence of EBV and *P. gingivalis* increases the risk of developing periodontitis and PI.

Further studies are necessary to establish the EBV as an etiological agent of PI. Prevention and new treatment could be developed as a strategy to keep latency of the EBV.

## Conclusions

The results from our study showed that coexistence of EBV and *P. gingivalis* was detected significantly higher in the PI patients than healthy controls and healthy implant patients. These results suggest that coexistence of EBV and *P. gingivalis* may serve pathogenic factors cause for PI in Japanese dental patients.

## Abbreviations

BOP: Bleeding on probing
CP: Chronic periodontitis
EBV: Epstein-Barr virus
GAPDH: Glyceraldehyde-3-phosphate dehydrogenase
HDAC: Histone deacetylase
PCR: Polymerase chain reaction
PI: Peri-implantitis
PM: Peri-implant mucositis
PPD: Probing pocket depth
SUP: Suppuration
